# Updated efficacy and safety of CDK4/6 inhibitors plus endocrine therapy in elderly women with HR+/HER-2 metastatic or advanced breast cancer: patient-level network meta-analysis

**DOI:** 10.18632/aging.206257

**Published:** 2025-05-25

**Authors:** Henry WC Leung, Mei-Ching Tsai, Shin-Hang Leung, Shyh-Yau Wang, Agnes LF Chan

**Affiliations:** 1 Department of Radiation Oncology, An-Nan Hospital, China Medical University, Tainan, Taiwan; 2 Department of Medical Materials Supply, Tainan Municipal Hospital (Managed by Show Chwan Medical Care Corporation), Tainan, Taiwan; 3 Department of Radiology, An-Nan Hospital, China Medical University, Tainan, Taiwan; 4 Department of Pharmacy, Kaohsiung Show Chwan Memorial Hospital, Kaohsiung, Taiwan

**Keywords:** breast cancer, elderly patients, abemaciclib, ribociclib, palbociclib, network meta-analysis

## Abstract

Background: Breast cancer (BC) is the most common cancer in women worldwide. More than 80% of new cases of invasive BC are diagnosed among women aged 50 years or older, and they mainly comprise estrogen receptor (ER)-positive and HER2-negative subtypes of the disease. About 91% of deaths occur in this age demographic. Treatment with cyclin-dependent kinase 4/6 inhibitors has resulted in significantly increased survival benefits in terms of progression-free survival and overall survival (OS), but evidence for their use in treating older women with metastatic BC is limited. Therefore, we evaluated the efficacy and safety of cyclin-dependent kinase 4/6 (CDK4/6) inhibitors combined with endocrine therapy in older women with HR+/HER-2 metastatic or advanced BC.

Methods: We conducted a comprehensive search of the PubMed and EMBASE databases between January 2018 and December 2024 for phase II or III randomized controlled trials (RCTs) investigating treatment modalities in HR+/HER-2 metastatic or advanced BC. Kaplan–Meier curves for progression-free survival (PFS) and overall survival (OS) were reconstructed to retrieve individual patient-level data to strengthen the comparison of the benefits of all treatment modalities of interest. In this network meta-analysis (NMA), each study was pooled in a fixed-effects or randomized-effects model based on the individual study quality. We also performed a subgroup analysis and reported the incidence of ≧grade 3 adverse events in elderly patients (≧65 years). The primary endpoints were the pooled PFS, OS, and comparable safety rankings. The treatment modalities were ranked using SUCRA scores.

Results: We identified 15 phase II and III randomized controlled trials with seven treatment modalities that met the inclusion criteria. From these trials, rates of PFS and OS for 1799 and 1568 patients, respectively, were included in the analysis. In terms of PFS, Palbociclib + Letrozole (Let) ranked highest among all treatment modalities, followed by Ribociclib + Fulvestrant (Ful). Meanwhile, Palbociclib plus Ful showed superior OS ranking compared to other treatments in older women with mBC. Regarding safety, Palbociclib plus Endocrine (letrozole or fulvestrant) (79.3%), Ribociclib plus Let (87%), and Abemaciclib + ET (letrozole or anastrozole) were associated with a relatively high incidence of ≧grade 3 adverse events (AEs) compared to placebo plus endocrine therapy.

Conclusions: In this network meta-analysis, the combination of Palbociclib with Letrozole or Fulvestrant was found to have an effect on PFS and OS, and Ribociclib + Let was found to be a relatively safe treatment option for elderly women with HR+/HER2 metastatic or advanced BC. However, given the limited evidence in older populations, comprehensive, well-designed, large-scale randomized controlled trials are needed to address this issue.

## INTRODUCTION

The elderly population is increasing worldwide, and the proportion of Taiwanese women who are ≧65 years old is expected to increase from 14% in 2018 to 20% in 2025 [1]. By 2024, approximately 310,720 women in the United States will be diagnosed with invasive breast cancer, with approximately two-thirds of cases occurring in women 55 years or older [2]. As the population ages and cancer incidence increases with age, cancer among the U.S. population aged 65 years or older is expected to increase by 67% from 2010 to 2030 [3]. Despite comprising a large proportion of breast cancer patients, older patients have historically been underrepresented in clinical trials of new anticancer drugs [4]. Due to this difference, clinical guidelines for the treatment of breast cancer in older adults are mainly based on evidence from younger patients, who may have different disease characteristics and prognoses. In addition, the treatment of elderly breast cancer patients is challenging because comorbidities and frailty often occur in advanced age [5].

Hormone receptor-positive (HR+)/human epidermal growth factor receptor 2-negative (HER2–) breast cancer is the most common molecular subtype among older patients and those with advanced/metastatic breast cancer (a/mBC). About 91% of deaths occur in this age demographic. Aging is one of the biggest risk factors for breast cancer in women. About 85% of breast cancers occur in women who have no family history of the disease. These cancers develop due to gene mutations caused by the aging process and life processes rather than inherited mutations [6]. Cyclin-dependent kinase 4/6 (CDK4/6) inhibitors have been shown to significantly increase survival benefits in terms of progression-free survival and overall survival (OS), but the evidence on treating older women with metastatic BC is limited. Therefore, we comprehensively evaluated the efficacy and safety of cyclin-dependent kinase 4/6 (CDK4/6) inhibitors combined with endocrine therapy (ET) in older women with HR+/HER-2 metastatic or advanced BC.

## MATERIALS AND METHODS

### Search strategy and selection criteria

We conducted a comprehensive search of the literature for phase II or phase III randomized controlled trials (RCTs) investigating the outcomes of anti-CDK4/6 inhibitors (CDK4/6i) combined with ET (Letrozole or Fulvestrant) versus ET alone in the treatment of elderly patients (65 years and older) with HR+/HER2− metastatic or advanced BC. The search for eligible randomized controlled trials (RCTs) was limited to studies published in English and available via the PubMed, Embase, Cochrane Library, and Web of Science databases, in accordance with the Preferred Reporting Items for Systematic Reviews and Meta-Analyses (PRISMA) guidelines [7]. The search included updated randomized controlled trials published between January 2018 and December 2023. The detailed search strategies are described in the Supplementary Table 1 (online only). From the selected articles, we manually searched for additional references to identify potentially overlooked studies.

All trials included met the following inclusion criteria: (1) they were randomized phase II or III clinical trials comparing anti-CDK4/6 inhibitors (Palbociclib, Ribociclib, or Abemaciclib) combined with ET (Letrozole or Fulvestrant) versus ET alone in the treatment of elderly patients (65 years and older); (2) they included patients with proven HR+/HER2− metastatic breast cancer or advanced BC; (3) they provided detailed data on their methods, the characteristics of the elderly patient population, overall survival, progression-free survival, and adverse events; and (4) they compared at least two groups containing the item of interest listed. Studies, case reports, case series, and reviews that did not meet the above inclusion criteria were excluded.

### Data extraction

Two reviewers independently reviewed and screened all eligible studies based on the above screening criteria. Any differences in opinion were evaluated by a third reviewer to achieve a consistent consensus. We extracted and summarized the characteristics of the population data from all eligible studies, including the name of each study’s first author, the intervention, and the outcome in terms of PFS, overall survival (OS), and SAEs (defined as ≧grade 3 AEs), in a standardized table. We also analyzed hematological and non-hematological subgroups in the SAEs.

We used individual patient data (IPD) analysis to produce more precise, reliable, and accurate results to compare the efficacy of the treatment modalities of interest based on patient characteristics. The advantage of utilizing IPD analysis is that it can enhance the clinical relevance of study results, resulting in more clinically meaningful analysis and interpretation. Therefore, we graphically reconstructed PFS and OS data from IPD in each trial arm, using WebPlotDigitizer to digitize the Kaplan–Meier (KM) curves based on the reconstructive algorithm outlined by Guyot et al. [8, 9]. Using visual comparison, log-rank p-values, and hazard ratios (HRs), we compared the reconstructed IPD PFS Kaplan–Meier plots and data with the values reported in the original studies. We did not reconstruct the OS KM curve because raw OS KM curve data were not available for older patients in every study included.

### Study quality assessment

Two independent investigators used the GRADE approach of the Cochrane Collaboration risk of bias (RoB) 2 assessment tool was used to assess the quality of each included study. There are five bias domains in the revised RoB tool [10], including (1) bias due to the randomization process, (2) deviation from intended intervention, (3) missing outcome data, (4) measurement of outcomes, and (5) selection of the reported result, as well as an “overall risk of bias” judgment. Each domain was explicitly evaluated as having a low risk of bias, a high risk of bias, or some concerns [11].

### Statistical analysis

Pairwise meta-analyses were performed using Review Manager Version 5.4, using the hazard ratio (HR) to estimate the pooled effect size [12]. Heterogeneity was assessed using the *I^2^* test, with *I^2^* values >50% indicating heterogeneity. If significant heterogeneity was observed, a random-effects model was selected; otherwise, a fixed-effects model was used. The results were reported as ORs with corresponding 95% confidence intervals (CIs). Statistical significance was defined as *P* < 0.05.

Bayesian network meta-analysis was performed using WinBUGS 1.4.3 (MRC Biostatistics Unit, Cambridge, and Imperial College School of Medicine, London, UK) and NetMetaXL (version 1.6.1) to compare the efficacy and safety of applying 6 CDK4/6 inhibitors plus endocrine therapy in elderly patients with HR+/HER-2 metastatic or advanced breast cancer [13]. In our network, a fixed-effects model and a random-effects model were determined based on the deviance information criteria (DIC). Model convergence was used to compare within- and between-chain variances to calculate the potential scale-reduction factor (PSRF). Convergence is good when the PSRF value approaches to 1.0 and the variations stabilize as the number of simulations increases [14]. Inconsistencies between direct and indirect evidence were assessed by plotting the posterior mean deviation for individual data points in the inconsistency model against the posterior mean deviation in the consistent model to identify potential inconsistencies within the network.

Cumulative ranking curves (SUCRAs) were used to rank treatments by summarizing and reporting the surface under the cumulative ranking curve, which is a summary of the rank distribution and can be interpreted as the estimated probability of the most effective treatment.

Sensitivity analysis was performed using fixed- and random-effects models to test the robustness of network comparisons by repeating the main calculations.

### Data availability statement

Data are provided within the manuscript or Supplementary Materials.

## RESULTS

### Characteristics of studies and patients included

By searching the PubMed, EMBASE, Cochrane Library, and Web of Science electronic databases, 15 randomized controlled trials involving seven treatment options were identified, with a total of 1799 and 1568 patients with PFS and OS results, respectively (Supplementary Figure 1). Among the studies, twelve were phase III randomized clinical trials [15–25], and three were phase II RCTs [26–28]. The publication years of the studies ranged from 2018 to 2024, with updated outcome data. Nine studies provided data on the HR for PFS and OS. However, data on the incidence of serious AEs are lacking, with only three studies reporting the percentage of AEs [1, 15, 25].

Most of the studies included patients aged 65 years and above, accounting for 42% to 70.3%. The majority of patients enrolled in the studies were postmenopausal women with HR-positive and HER2-negative recurrent or metastatic breast cancer who had not received prior systemic therapy for advanced disease. Viscera and bone were the most common sites of metastasis, ranging from 48.7% to 60% and 13.8% to 75.6%, respectively. In most studies, patients aged >65 years had an ECOG performance status of 0 or 1. The study and patient characteristics are presented in Table 1.

**Table 1:** Characteristics of studies included >65 yrs patients in the analysis.

**Author/year**	**Study type**	**≧65 yrs ages, Events, n/N, %**	**Intervention Group (A)**	**Comparator Group (B)**	**≧Gr 3 neutropenia (%) A vs. B**	**Any Grade AE A vs. B (%)**	**HR PFS (95%CI)**	**HR OS (95%CI)**	**Site of metastases, *n*%**		
									**Visceral^a^**	**Bone**	**LN**
1. Sonke/2018 [14] 2. Hortobagyi/2022 [15] (MONALEESA-2)	Phase 3	150/295 (51) 145/295 (49)	Ribociclib + Letrozole	Letrozole + placebo	78% 26%	99% 94%	0.60 (0.39−0.92)	NA 0.87 (0.64−1.18)	176 (59.7) 164 (55.7)	216 (73.2) 65 (22.2)	NA
3. Slamon/2018 [16] (MONALEESA-3)	Phase 3	95/226 (42.0) 70/113 (61.9)	Ribociclib + Fulvestrant	Ful + placebo	NA	NA	0.597 (0.436−0.818)	NA	293 (60.5)	103 (75.6)	NA
4. Slamon/2021 [17] (MONALEESA-3)	Phase 3	106/226 (46.9) 67/113 (59.3)	Ribociclib + Fulvestrant	Ful + placebo	–	–	–	0.72 (0.53−0.99)	170 (50)	71 (21)	NA
5. Sledge/2020 [18] (MONARCH-2)	Phase 3	52/92 (56.5) 86/155 (55.5)	Abemaciclib + Ful + placebo	Ful + placebo	NA	NA	0.63 (0.45-0.87)	0.898 (0.638−1.263)	137 (55.9)	66 (26.9)	NA
6. Goetz/2021 [19] (MONARCH-3)	Phase 3	52/90 (57.8) 86/155 (55.4)	Abemaciclib + Ful + placebo	Ful + placebo	54% (163/302) 7.4% (12/162)	65% 72%	0.60 (0.47−0.77)	NA	119 (53.6)	58 (23.7)	NA
7. Goetz/2024 [20] (MONARCH-3)	Phase 3	49/74 (66.2) 93/140 (66.4)	abemaciclib plus NSAI	NSAI + placebo			–	0.751 (0.539−1.049)	118 (53)	49 (22.0)	NA
8. Zhang/2020 [21] (MONARCH PLUS)	Phase 3	A:16/50 (32.0) 9/16 (56.3) B: 12/26 (46.2) 6/14 (42.9)	A:Abemaciclib + NSAI B: Abemaciclib + Ful+ placebo	NSAI + Placebo Ful + placebo	NA	NA	2.50 (0.56−11.1) 0.56 (0.24−1.26)	NA-	24 (60)	NA	NA
9. Slamon/2024 [22] (PALOMA-2)	Phase 3	111/181 (61.3) 50/81 (61.7)	Palbociclib + Letrozole	Letrozole + placebo	NA	NA		0.87 (0.62−1.22)	127 (48.5)	NA	NA
10. Rugo/2018 [23] (PALOMA 3)	Phase 3	39/86 (45.3) 22/43 (51.2)	Palbociclib + Ful + placebo	Ful + placebo	NA	NA	0.31 (0.19-0.50)	NA	150 (49.3)	63(20.7)	NA
11. Xu/2022 [24] (PALOMA-4)	Phase 3	11/14 (78.6) 18/24 (75.0)	Palbociclib + Letrozole	Letrozole + placebo	NA	NA	1.247 (0.585−2.657)	NA-	21 (55.3)	NA	NA
12. Turner 2018 [25] (PALOMA-3)	Phase 3	33/86 (38.4) 17/43 (39.5)	Palbociclib + Ful + placebo	Placebo + Ful	NA	NA	NA	0.52 (0.33−0.82)	311-(59.7)	NA	NA
13. Albanell/2021 [26] (FLIPPER)	Phase 2 Random	20/47 (42.3) 27/45 (60.0)	Palbociclib+ Ful + placebo	Ful + placebo	NA-	NA-	0.51 (0.34−0.75)	NA-	55 (59.8)	62 (67.4)	NA
14. Rugo/2018 [27] 15. Finn/2020 [28] (PALOMA-1)	Phase 2 open-label	91/218 (41.7) 78/120 (65.0) 27/39 (69.2) 26/37 (70.3)	Palbociclib + Letrozole Palbociclib + Letrozole	Letrozole + placebo Letrozole + placebo	79% vs. 24%	99% 93%	0.55 (0.39−0.76)	0.97 (0.57−1.65)	37 (48.7)	13 (18.6)	50 (54.3)

^a^Includes liver, Lung. Abbreviations: HR: hazard; PFS: progression-free survival; OS: overall survival; NSAI: non-steroidal aromatase; Ful: Fulvestrant; LN: Lymph nodes.

### Network meta-analysis of treatment effects

#### Pairwise analysis

In terms of PFS, the efficacy of CDK 4/6 inhibitors plus ET (Letrozole or Fulvestrant) presented as HR in pairwise analysis was 0.56 (95% CI: 0.50, 0.63) (Supplementary Figure 2).

#### Kaplan-Meier survival curve in NMA

**Figure 1:**
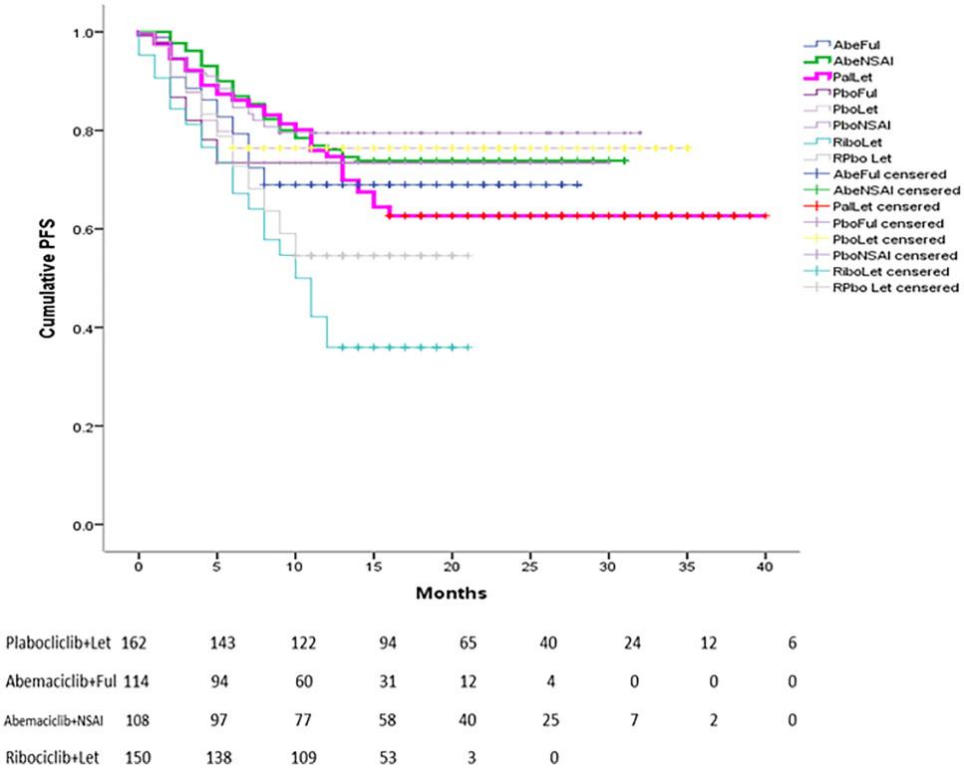
Reconstructed Kaplan-Meier curves of PFS for individual patient data extracted from original studies related to CDK 4/6 for advanced BC.

Palbociclib/Letrozole was found to be comparable to Ribociclib/Fulvestrant (OR =0.96, 95% CI=0.51 – 1.84) and significantly superior to Abemaciclib/Ful (OR= "0.46;95% CI=0.23 – 0.89), Ribociclib/ Let (OR = 0.35, 95% CI = 0.17 – 0.69) and placebo plus Let or Ful (OR=0.43, 95% CI=0.27 – 0.67). If compared with Abemaciclib+NSAI and Palbociclib+Ful, no significant difference was observed in terms of PFS. In addition, no significant improvement in OS was found for all combinations of CDK4/6i with ET or NSAI (Table 2). A Kaplan–Meier pooled comparison plot of PFS for each regimen is shown in Figure 1.

**Table 2:** The league table for comparisons of progression survival (PFS) and overall survival (OS).

**Palbociclib+Let**	0.74 (0.40–1.37)	0.97 (0.45–2.07)	0.61 (0.29–1.32)	0.87 (0.42–1.79)	0.97 (0.61–1.56)	0.70 (0.36–1.34)
0.96 (0.51–1.84)	**Ribociclib+Ful**	0.76 (0.38–1.57)	0.83 (0.50–1.35)	0.65 (0.34–1.25)	0.76 (0.52–1.10)	094 (0.51–1.69)
0.67 (0.34–1.33)	0.70 (0.35–1.38)	**Abemaciclib+NSAI**	0.63 (0.27–1.47)	0.84 (0.37–1.89)	1.00 (0.54–1.81)	0.71 (0.34–1.54)
0.67 (0.33–1.37)	0.70 (0.34–1.45)	1.00 (0.48–2.13)	**Palbociclib+Ful**	0.54 (0.24–1.19)	0.63 (0.35–1.13)	0.88 (0.42–1.87)
0.46 (0.23–0.89)	0.47 (0.24–0.92)	0.68 (0.34–1.38)	0.68 (0.32–1.42)	**Abemaciclib+Ful**	0.85 (0.49–1.47)	0.61 (0.30–1.24)
0.43 (0.27–0.67)	0.44 (0.28–0.70)	0.64 (0.38–1.06)	0.64 (0.37–1.11)	0.94 (0.57–1.53)	**Placebo+Let/Ful**	0.72 (0.45–1.13)
0.35 (0.17–0.69)	0.36 (0.18–0.72)	0.52 (0.25–1.08)	0.52 (0.24–1.11)	0.77 (0.37–1.56)	0.82 (0.49–1.37)	**Ribociclib+Let**

Data are presented as odds radio (OR) and 95% confidence intervals (CI). For PFS, OR < 1 favors the treatment on left column; For OS, OR < 1 favors the treatment on left row. Yellow color presented PFS and green color was OS.

#### The SUCRA score plot ranking

**Figure 2:**
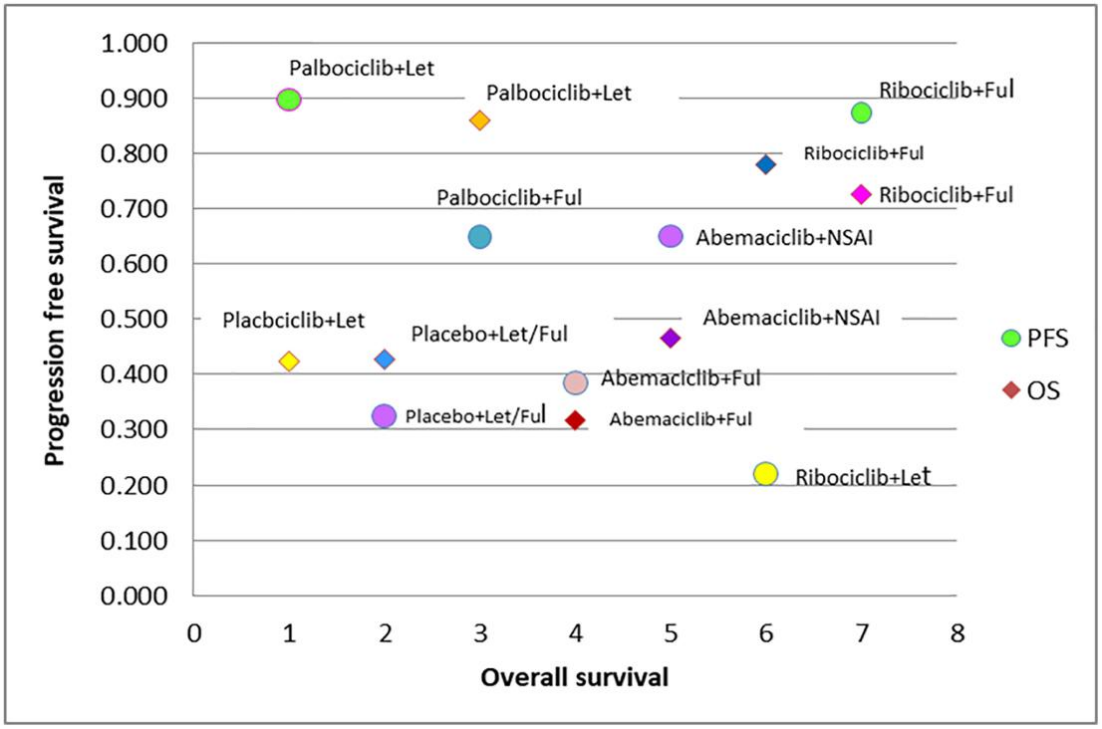
SUCRA plot of CDK 4/6 efficacy ranking for progression free survival vs. overall survival.

The SUCRA score plot for PFS versus OS is shown in Figure 2. The probability rankings of the seven treatments show that Palbociclib+Let was associated with higher PFS but lower OS, while Ribociclib+Fulvestrant was associated with higher PFS and OS. The PFS- and OS-improving effects of Ribociclib+Fulvestrant ranked high. However, the probability ranking of Ribociclib plus Let was lower in PFS but higher in OS, while the results for the other regimens were comparable (Figure 2).

### Network meta-analysis: treatment safety

Serious adverse events (≥ grade 3) are described in Table 3. In the fifteen studies included, there are only three reported adverse events in patients aged ≥ 65 years. The overall incidence of serious adverse events ≥ grade 3 was 53.9% to 79.3%. Palbociclib+EN were associated with relatively higher all-grade AEs and grade ≥3 severe AEs compared with Ribociclib+Let or Abemaciclib+ET.

**Table 3:** Treatment-related adverse events occurring in ≧10% of patients ≧65 years in either treatment group.

**Authors study**	**Intervention Comparator**	**No of patients**	**Any AE (*N*) %**		**AEs ≧Grade 3 (*N*) %**									
AE, *n* (%)^*^		**≧65**	**All Grade**	**Grade ≧3**	**Hematology**					**Non-Hematology**				
					**Neutropenia**	**Leucopenia**	**Anemia**	**HTN**	**Diarrhea**	**ALT increased**	**AST increased**	**Fatique**	**Infection**	**VTE events**
Rugo 2018	Pabociclib+EN	304	302 (33.4)	241 (79.3)	201 (66.1)	80 (26.1)	17 (5.6)	–	5 (1.64)	–	–	13 (4.3)	26 (8.6)	–
Phase II RCT	Placebo+EN	161	150 (93.2)	33 (24.2)	1 (0.62)	0	3 (1.86)		0 (0.0)			0	6 (3.7)	
Snoke 2018	Ribociclib+Let	150	148 (99)	130 (87)	90 (60)	31 (21)	2 (1)	23 (15)	3 (2)	14 (9)	6 (4)	3 (2)	–	–
MONALEESA-2	Placebo+Let	144	139 (97)	56 (39)	0	1 (1)	2 (1)	25 (17)	1 (1)	0	3 (2)	2 (1)		
Phase III RCT														
Goetz 2021	Abemaciclib+ET	302	100 (33.1)	163 (53.9)	75 (24.8)	–	–	–	44 (14.5)	16 (5.3)	9 (3.0)	–	–	10 (3.3)
MONARCH-2,3	Placebo+ET	162	19 (11.7)	47 (29.0)	2 (1.2)		–		2 (1.23)	2 (1.2)	4 (2.5)			1 (0.62)
Phase III RCT														

^*^Pooled TEAEs from MONARCH 2 and MONARCH 3 occurring in ≥10% of patients in any age group. Abbreviations: NSAI: non-steroidal aromatase inhibitor; EN: endocrine therapy; ET: letrozole or anastrozole; − means not available.

In the subgroup analysis of the incidence of hematological SAEs ≧ grade 3 (including neutropenia, leukopenia, and anemia), the results for Pabociclib+EN (66.1%, 60.0%, and 24.8%, respectively) were higher than for Ribociclib+Let and Abemaciclib+ET. In terms of non-hematological aspects, the incidence of diarrhea was higher in the group treated with Abemaciclib+ET (28.8%) compared to the other two regimens. Furthermore, in the subgroup analysis, a higher rate of incidence of venous thrombosis events (VTEs) was observed only in patients aged ≥75 years who received Abemaciclib+ET.

### Study quality assessment

Good quality was observed in the included studies because no heterogeneity between studies (*p* = 0.06, I^2^ = 40%) was identified via pairwise meta-analysis (Supplementary Figure 2).

**Figure 3:**
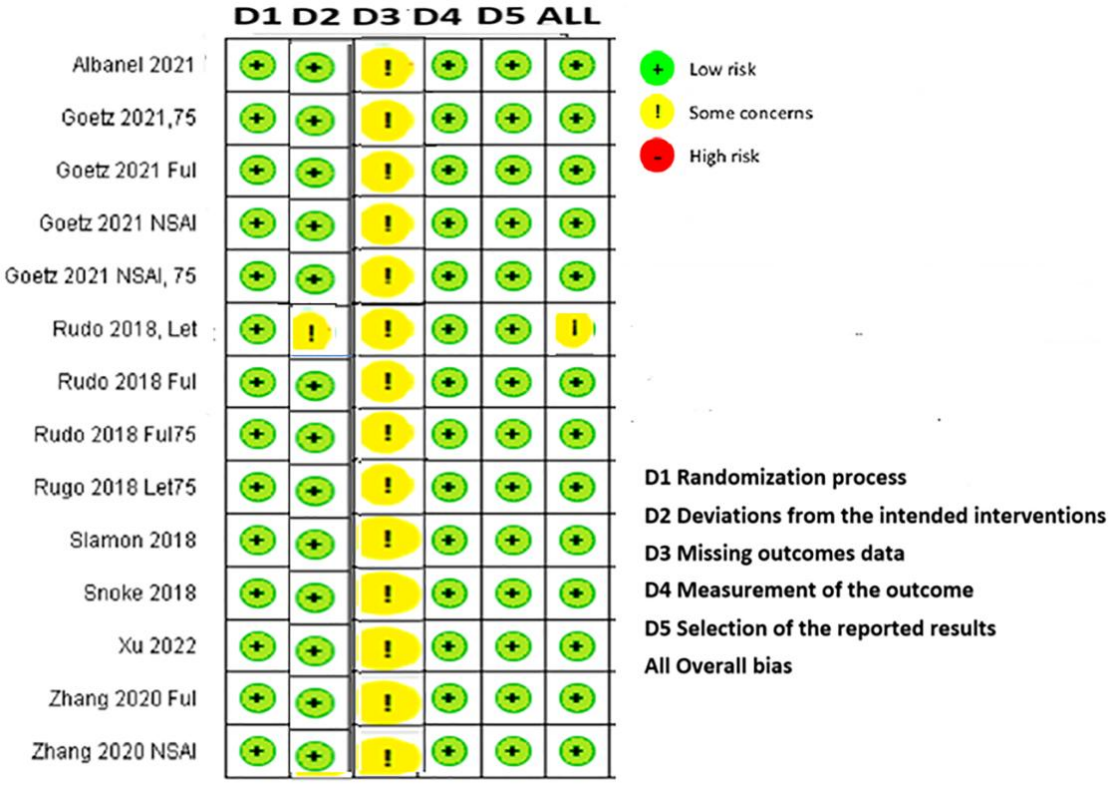
Cochrane risk-of-bias assessment tool for randomized trials version 2.

The results obtained using the RoB2 tool indicated that all identified RCTs had a low risk of bias in four domains [28]. Although some concerns were raised about missing outcome data in all included studies [15–27] and deviations from the intended intervention domain in one phase II open-label study [24], all overall bias domains except PALOMA-1 were determined to be low-risk according to both raters (Figure 3).

No evidence of inconsistency between direct and indirect evidence impacted the outcomes of this network meta-analysis because the individual data points’ posterior mean deviance contributions for the consistency model (horizontal axis) and the unrelated mean effects model (vertical axis) followed the line of equality. Therefore, assessment inconsistency did not affect the results of the network meta-analysis (Supplementary Figure 3).

Sensitivity analysis results for the network comparisons showed no significant difference between the random- and fixed-effects models for PFS (Tau = 0.1269; 95% CrI: 0.03872–0.3873 and OS (Tau = 0.138; 95% CrI: 0.04282–0.4189). The results of this study are robust.

## DISCUSSION

To the best of our knowledge, this study describes the first NMA using data from phase II or III RCTs to compare the efficacy and safety of three CDK4/6 inhibitors in elderly patients with HR+/HER2− metastatic or advanced breast cancer. According to epidemiological studies, over two-thirds of patients diagnosed with HR+/HER2- breast cancer are aged 65 years or older [29–31]. Therefore, due to the high prevalence of pre-existing comorbidities and the perceived severity of the risks of using more aggressive conventional treatments in elderly individuals, the novel target therapy is considered appropriate for older patients.

Based on our findings, it can be concluded that the addition of CDK4/6 inhibitors to treatment for elderly women with HR+/HER2 subtype metastatic or advanced BC can significantly and non-significantly improve PFS and OS with a similar incidence rate of severe AEs. The combinations of Palbociclib plus Letrozole and Ribociclib plus Letrozole were considered to have a greater effect on PFS and OS and a comparatively safe treatment option for this patient group. This result was supported by a systematic review and meta-analysis of retrospective real-world data on the use of CDK4/6 inhibitors in older and younger patients with breast cancer, indicating that Palbociclib and Ribociclib provided a better survival benefit to elderly patients [32].

Our findings reflect the difference of the patient selection in clinical trials and retrospective real-world. The general differences included the age limit to 65 years for older patients and relatively healthy. In contrast, retrospective studies have defined higher cutoff values for the elderly population aged ≥70 years. Although they had significantly more dose reductions and dose delays than younger patients, their PFS improvement is still significantly comparable with younger patients. Those studies reported the outcomes were not related to age [33]. In another real-world series, Wilkie et al. found no significant difference in dose reductions among women older and younger than 70 years [34]. The HR for PFS of the combination of CDK 4/6 inhibitors + aromatase inhibitors (AI) compared with AI alone was 0.52 (95% CI, 0.38 to 0.70) in an FDA pooled analysis [35], which was similar to the result attained in our analysis with patients older than 65 years (HR = 0.56 (95% CI: 0.50, 0.63)). In addition, our results are also supported by a recently published real world retrospective study, which indicated that patients aged 70 experienced prolonged PFS in response to CDK4/6 inhibitor-based therapy, particularly when combined with AI [36].

Recently, breast cancer in geriatric patients is considered a global challenge in the upcoming decades due to the ageing of the population worldwide. Many studies report that breast cancer has increased in elderly patients (≥70 years) and suggest that the higher cancer mortality in this population could be related to organ dysfunction, an advanced and delayed diagnosis, and other morbidities. Furthermore, the elderly population with mBC remains excluded from clinical trials. Therefore, few data on the efficacy, safety, and short- and long-term outcomes of therapies based on the combined treatment of chemotherapy with CDK4/6 inhibitors are available. The results of this study can serve as updated evidence to confirm the efficacy and safety of CDK4/6 inhibitors in elderly patients with advanced or metastatic breast cancer.

Regarding safety, there were limited data reporting severe AEs in elderly patients in the included studies, but the incidence of severe AEs of grade 3 or above identified in the pooled analysis of included RCTs was relatively higher for Pabociclib+EN than for Ribociclib+Let and Abemaciclib+ET. In the subgroup analysis, neutropenia and diarrhea grades (G) 3–4 were similar in elderly patients. This result was consistent with a recently published systematic review and meta-analysis [32]. In addition, SAEs of VTE were reported only in patients aged ≥65 years treated with Abemaciclib+ET in our NMA. This result was consistent with previously published RCTs showing that VTE is an adverse event of particular concern with abemaciclib and is more common in patients aged ≥75 years old [37]. Another recently published review and meta-analysis reported that patients treated with Abemaciclib+ET had a higher VTE rate than those treated with Pabociclib+EN and Ribociclib+Let [38]. Therefore, these patients should be monitored more carefully for early symptoms during treatment tumor cells, immune cells, mesenchymal cells, cancer-linked fibroblasts, and extracellular matrix.

With the advancement of biotechnology and continuous research on the pathogenesis of breast cancer, the tumor microenvironment, comprising cellular components (such as cancer-associated fibroblasts, immune cells, endothelial cells, and adipocytes) and noncellular components (such as the extracellular matrix, cytokines, chemokines, signal molecules) has been recognized as a critical contributor to the development and progression of BC [39, 40]. The present hypothesis is that interactions between TME components and cancer cells promote phenotypic heterogeneity, cellular plasticity, and cancer cell stemness, leading to tumor dormancy, enhanced invasion and metastasis, and the development of therapeutic resistance [41]. While previous research focused on targeting cancer cells with a poor prognosis, novel therapies targeting stromal components are currently under preclinical and clinical investigation. The efficacy of TME-guided therapy when used alone or in combination with chemotherapy or radiotherapy, tumor staging or the identification of molecular features and novel breast cancer stage-specific biomarkers, will help determine precise TME-guided therapy. In particular, adipocytes and fibroblasts become especially rich in elderly mammary glands [42].

In an era of population ageing, clinical decisions should be optimized based on several factors rather than the patients’ age alone. These factors relate to the patients’ comorbidities, performance status, life expectancy, and pathological tumor and molecular characteristics. Appropriate geriatric assessment is extremely important, yet healthcare providers do not embrace this process in an effort to avoid unnecessary under-treatment or subjecting patients to intolerable toxicity. The introduction of “geriatric oncology” as a specialty with proper focused training for oncologists across all field of oncology will hopefully improve the care of this very vulnerable group of patients.

The strength of this study is the low risk of bias in the randomization process in the NMA because all studies included provided the latest published data from phase II and III randomized clinical trials. Furthermore, individual patient data were used to enhance the accuracy of the results. However, this analysis had several limitations. First, adverse event data were not presented in all studies included; therefore, the toxicity of the treatment regimens may be underestimated. Second, geriatric comorbidities and geriatric assessments were not recorded or extensively assessed in any study, so publication bias may be present in this analysis.

Third, patients participating in RCTs are generally healthier than those in real-world retrospective studies, which may affect the accuracy of this NMA by excluding frail patients encountered in clinical practice. Despite these limitations, the sensitivity analysis was robust. This study provides clinicians with the first updated pooled estimates of CDK4/6 inhibitor efficacy and safety in elderly patients with advanced BC, providing further evidence for their clinical application.

## CONCLUSIONS

Overall, this is the first pooled analysis to demonstrate the OS and PFS benefits of CDK4/6 inhibitors in elderly patients (age ≥65 years) with advanced HR+/HER-2 metastatic or advanced breast cancer. We recommend that the National Health Administration in our country reinforce physicians’, nurses’, and caregivers’ training on clinical breast self-examination and continue to promote mammography screening for elderly patients. Efforts should also be made to educate health professionals on the importance of conducting an appropriate assessment of the health status of older patients with cancer by using validated instruments and geriatric assessment tools. This assessment should be discussed with and offered to all patients after geriatric assessment and according to their toxicity profile. Finally, oncogeriatric assessment should be systematically considered if accessible.

## Supplementary Materials

Supplementary Figures

Supplementary Table 1
