# Supplementary material for: Updated efficacy and safety of CDK4/6 inhibitors plus endocrine therapy in elderly women with HR+/HER-2 metastatic or advanced breast cancer: patient-level network meta-analysis
Source: Aging (Albany NY). 2025 May 25;17(5):1313–27. doi: 10.18632/aging.206257 (PMC12151516; doi:10.18632/aging.206257)
Supplement: Supplementary Figures [file aging-17-206257-s001.pdf]

SUPPLEMENTARY FIGURES

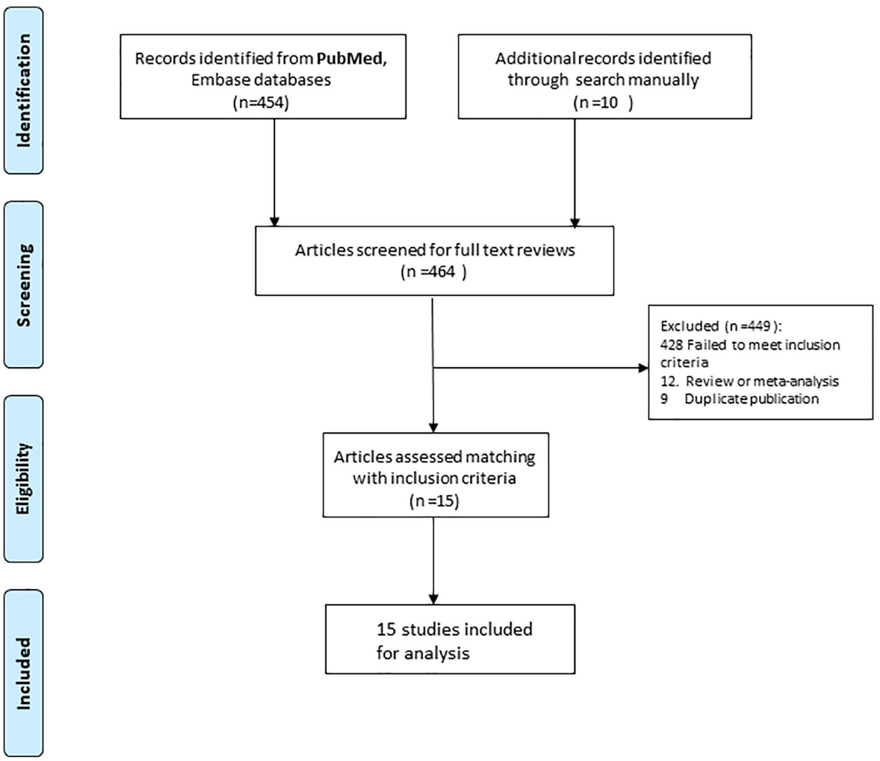

Supplementary Figure 1. The flow chart summarizing the process for the identification of the eligible clinical studies.

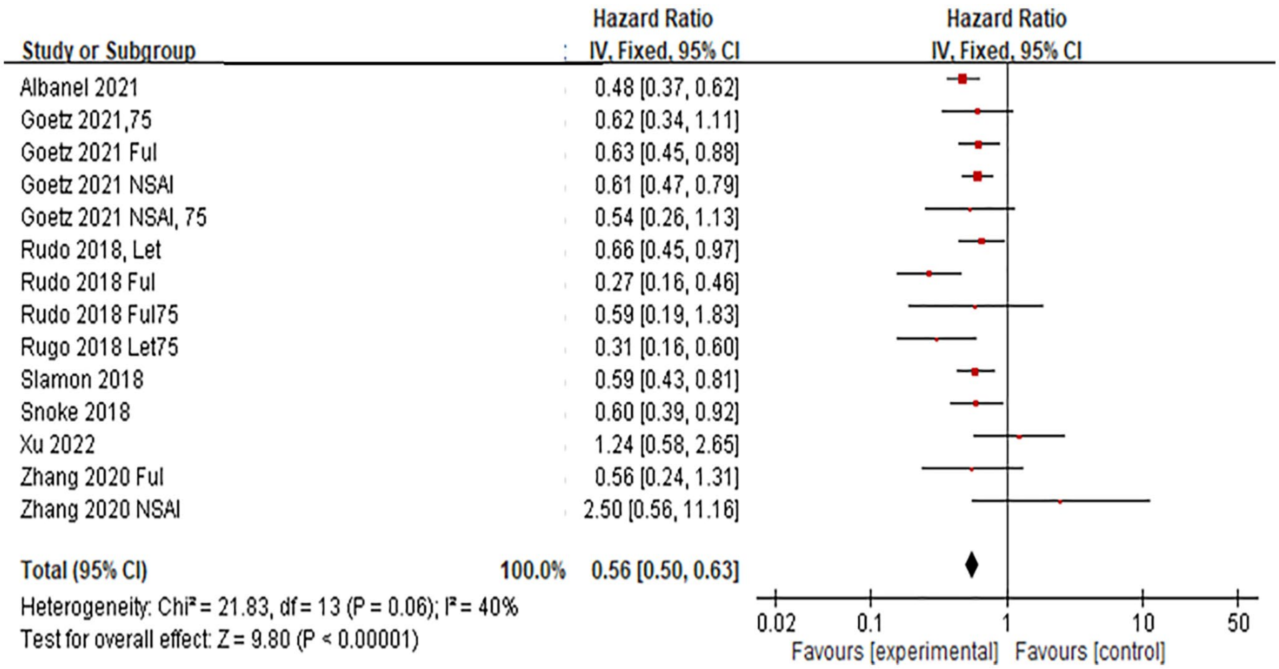

Supplementary Figure 2. PFS in pairwise analysis.

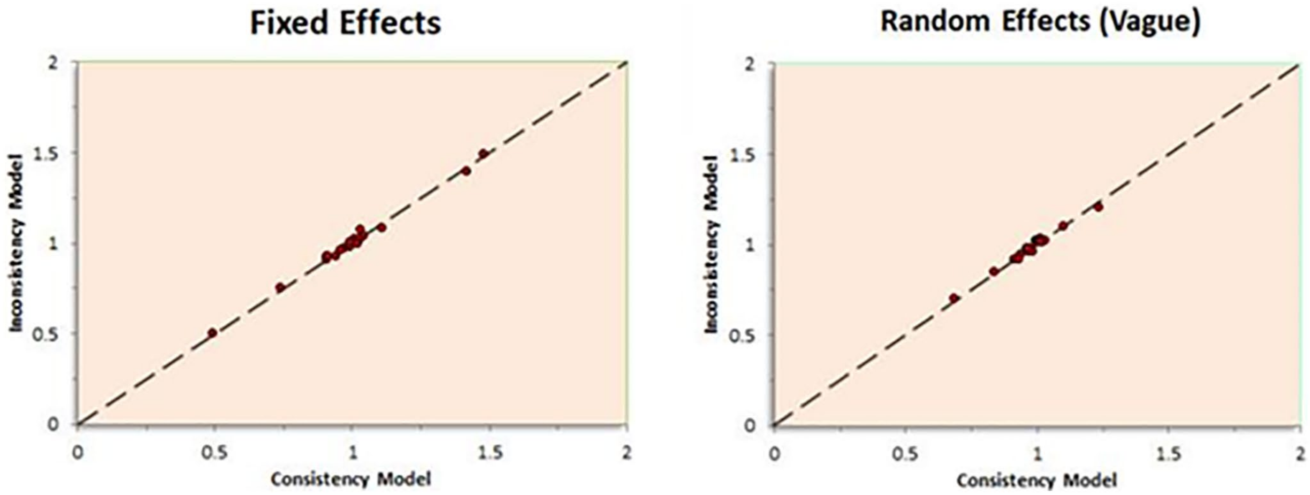

Supplementary Figure 3. Inconsistency vs. consistency plot for Progression free survival of COK 4–6 treatments. Plot of individual data points for the consistency model (horizonal axis) and the inconsistency model (vertical axis), along with the equality line.
