# Supplementary material for: Updated efficacy and safety of CDK4/6 inhibitors plus endocrine therapy in elderly women with HR+/HER-2 metastatic or advanced breast cancer: patient-level network meta-analysis
Source: Aging (Albany NY). 2025 May 25;17(5):1313–27. doi: 10.18632/aging.206257 (PMC12151516; doi:10.18632/aging.206257)
Supplement: Supplementary Table 1 [file aging-17-206257-s002.pdf]

SUPPLEMENTARY TABLE

Database: PubMed (June 23, 2024)

Search strategy	Number of records
1. Palbociclib plus endocrine therapy	190
2. old age or elderly AND advanced breast cancer	287
3. 1+2	35
4. ribociclib plus endocrine therapy	85
5. 4+2	17
6. Abemaciclib plus endocrine therapy	90
7.6+2	13
8. Phase III randomized clinical trial	31,572
9. 6+2+8	6
10. 4+2+8	11
11. 1+2+8	11

Database: Embase (June 23, 2024)

Search strategy	Number of records
1. ('palbociclib plus endocrine therapy':ab,ti AND 'old age':ab,ti OR elderly:ab,ti) AND 'advanced breast cancer':ab,ti AND 'phase 3 clinical trial':ab,ti	6
2. ('ribociclib plus endocrine therapy':ab,ti AND 'old age':ab,ti OR elderly:ab,ti) AND 'advanced breast cancer':ab,ti AND 'phase iii':ab,ti	15
3. ('abemaciclib plus endocrine therapy':ab,ti AND 'old age':ab,ti OR elderly:ab,ti) AND 'advanced breast cancer':ab,ti AND 'phase 3':ab,ti	6
4. #1 or #2 or # 3	6

Database: Cochrane Library (Mar 15, 2025)

Search strategy	Number of records
# 1. Palbociclib plus endocrine therapy):ti,ab,kw	230
# 2. elderly or old women or female patients):ti,ab,kw	32806
# 3. metastatic or advanced breast cancer	20287
# 4. #1 AND # 2 AND # 3	191
# 5. (Ribociclib plus Fulvestrant):ti,ab,kw (Word variations have been searched)	49
# 6. #5 AND # 2 AND # 3	42
# 7. Abemaciclib plus letrozole or anastrozole	466
# 8. #7 AND #2 AND #3	163
#9. Lerociclib plus fulvestrant	3
#10. #9 AND #2 AND #3	3

Database: Web of Science (March 15, 2025)

#1. TITLE (“CDK4/6”) AND (“elderly patient or women”) AND (“Phase III randomized clinical trial”) AND (“metastatic or advanced breast cancer”)	0
#2. TITLE (“Palbociclib plus letrozole or fulvestrant “) AND (“Ribociclib plus fulvestrant “) AND (“Abemaciclib plus letrozole or fulvestrant “) AND (“elderly patient or women”) AND (“Phase III randomized clinical trial”) AND (“metastatic or advanced breast cancer”)	0
